# DNA double strand break repair pathway choice: a chromatin based decision?

**DOI:** 10.1080/19491034.2015.1010946

**Published:** 2015-02-12

**Authors:** T Clouaire, G Legube

**Affiliations:** 1 Université de Toulouse; UPS; LBCMCP; Toulouse, France; 2 CNRS; LBCMCP; Toulouse, France

**Keywords:** chromatin, trimethyl H3K36, DNA double strand breaks, DSB pathway choice, homologous recombination

## Abstract

DNA double-strand breaks (DSBs) are highly toxic lesions that can be rapidly repaired by 2 main pathways, namely Homologous Recombination (HR) and Non Homologous End Joining (NHEJ). The choice between these pathways is a critical, yet not completely understood, aspect of DSB repair. We recently found that distinct DSBs induced across the genome are not repaired by the same pathway. Indeed, DSBs induced in active genes, naturally enriched in the trimethyl form of histone H3 lysine 36 (**H3K36me3**), are channeled to repair by HR, in a manner depending on SETD2, the major H3K36 trimethyltransferase. Here, we propose that these findings may be generalized to other types of histone modifications and repair machineries thus defining a “DSB repair choice histone code”. This “decision making” function of preexisting chromatin structure in DSB repair could connect the repair pathway used to the type and function of the damaged region, not only contributing to genome stability but also to its diversity.

## Homologous Recombination and Non Homologous End Joining Can Both Drive Genome Instability

DSBs are repaired either by Homologous Recombination (HR) or Non Homologous End Joining (NHEJ) mechanisms. Defects in either repair pathway results in genome instability and can be lethal at very early developmental stages. Although complementary, these mechanisms are markedly different (reviewed in^1^). The homology driven repair (HR), relies on extensive resection at the DSB to generate single strand DNA that will invade an intact copy of the damaged locus and use it as a template. In contrast, NHEJ repair machineries trigger no or limited resection and are able to join the 2 broken ends, with no or minimal homology.

Importantly failure or misuse of each of these DSB repair pathways can trigger very different consequences on the genome. Classical NHEJ (C-NHEJ), although mainly conservative, can occasionally be associated with point mutations and small deletions, depending on the structure of the DNA ends. Microhomology Mediated End Joining (an alternative NHEJ pathway) leads to the deletion of the sequence between microhomologies (reviewed in^2^). In addition, recent studies suggest that NHEJ is the primary cause of translocations^3,4^ and dysfunctional telomeres fusion.^5^ Finally, while HR pathways can be entirely conservative when the sister chromatid is used as a template, dramatic events such as repeat amplification/deletion or loss of heterozygosity (LOH) can occur when HR operates on repeated sequences or homologous chromosomes, respectively. As an example, the improper use of unequal homologous recombination to repair rDNA (rDNA) leads to the production of extrachromosomal rDNA circle (ERC), believed to be toxic for cells and associated with aging.^6,7^ Increased recombination on rDNA has even been proposed to be a molecular basis of aging in both yeast and higher eukaryotes (for review see,^8^) as well as a potential driving force of genomic instability in cancer cells.^9^

## DSB Repair Pathway Choice is Critical

Clear evidence suggests that both HR and NHEJ can co-exist in the same cell.^10^ In addition, the loss of either one of these pathways can be compensated for in most cells by another DSB repair mechanism, indicating that they compete to some extent for repair of a defined DSB. Given the distinct consequences that arise from these repair mechanisms at both sequence and chromosomal levels (see above), the choice between C-NHEJ, Alt-NHEJ/MMEJ, and the various HR-related pathways to repair a defined DSB is with no doubt a critical aspect of DSB repair (for review^11^). However, how this choice is performed is far from understood. Mechanisms that have been proposed to participate in this decision include cell type, age of the cell and cell cycle phase, as well as the persistent nature and complexity of a break. Many recent studies have put effort into identifying the key factors that could regulate the switch toward one or the other pathway. For example, factors inhibiting resection such as the Ku heterodimer,^12^ 53BP1 and its effectors RIF1 (reviewed in^13^) or PTIP^14^ will favor NHEJ. On the other hand, CtIP^15^ and the MRE11 nuclease^16^ promote resection and thus HR. But how these proteins compete for access to a specific break to achieve their function in DSB repair pathway choice is still uncovered.

## Function of H3K36me3 in DSB Repair Pathway Choice

In eukaryotes, DNA associates with various proteins, mainly histones, to form chromatin, which tightly regulates its accessibility and therefore plays a key role in DNA metabolism. Chromatin is a highly dynamic structure, affected by multiple mechanisms, such as histone post-translational modifications (i.e. methylation, acetylation, phosphorylation…), DNA methylation, incorporation of histone variants or local nucleosome density.

Genome-wide mapping of histone modifications and chromatin protein occupancies have shown that the genome is divided into distinct functional chromatin “states”, broadening the classical distinction between euchromatin and heterochromatin (for review^17^). Indeed, specific combinations of histone modifications are diagnostic of different genomic features (such as promoters, enhancers, gene bodies, insulators, transposons) as well as their regulatory states (e.g., actively transcribed, silenced or poised). A paradigmatic example is H3K36me3, which associates with transcription elongation and accumulates over the body of actively transcribed genes.^18^ Effector proteins will be able to translate such “chromatin signatures” to functional outcomes based on their ability to “read” modified histones by means of a highly specialized protein domain. For example, methylated histone tails can be recognized by Chromo-, PHD or Tudor domains, while Bromodomains or BRCT domains can respectively interact with acetylated or phosphorylated histones.^19^

Such chromatin patterns are very tightly linked with cell identity and disease state. Hence, huge efforts have been made to connect chromatin signatures with specific patterns of gene expression. Interestingly, beyond the relation between chromatin and transcription, recent data are in line with the attractive hypothesis that a pre-established chromatin structure could also play an instructive, “decision making” role in addressing adequate DNA repair pathways depending on where a DSB occurs in the genome. This structural adaptation would permit to use the most suited pathway, in terms of efficiency, accuracy and potentially deleterious outcomes, to repair a specific locus.

Using a human cell line (called DIvA for DSB Inducible via *Asi*SI) in which multiple annotated DSBs can be induced in a controlled manner using a restriction enzyme,^20, 21^ we established that distinct DSBs across the genome are not necessarily repaired by the same pathway.^22^ By ChIP-seq mapping of XRCC4 (a NHEJ component) and RAD51 (involved in HR), we identified an HR-prone subset of AsiSI-induced DSBs that, during the G2 cell cycle phase, is able to recruit RAD51, undergo resection and rely on RAD51 for efficient repair, and a non-HR prone subset, that even in G2 is unable to recruit RAD51 and require XRCC4 for efficient end joining. In agreement with the idea that repair pathway choice depends on the functional properties of the damaged locus, we found that HR-prone DSBs are located in actively transcribed genes and repair at such DSBs can be switched to RAD51-independent repair pathway upon transcriptional inhibition. Conversely, transcriptional activation favored RAD51 recruitment to an otherwise non-HR prone DSB.

**Figure 1.**
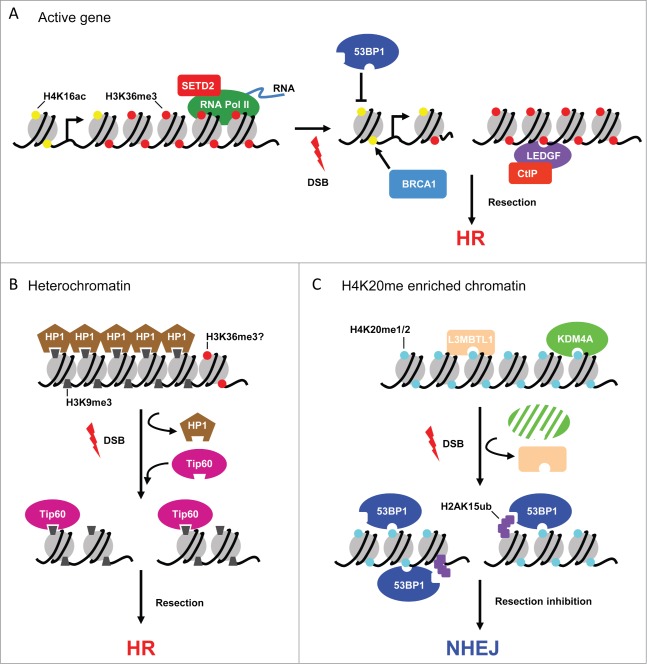
Preexistent chromatin structure influences DSB repair. (**A**) Actively transcribed genes are enriched for the transcription elongation mark H3K36me3 (red circles) by the action of SETD2 associating with elongating RNA Pol II. Histone acetylation on H4K16 (H4K16ac, yellow circles) is also enriched on active transcription units. Upon DSB induction, LEDGF-mediated CtIP recruitment, via H3K36me3 recognition, will favor resection and channel repair toward HR. H4K16ac will impede 53BP1 accumulation near DSBs, which will favor resection and BRCA1 dependent repair. (**B**) Condensed heterochromatin is characterized by H3K9me3 enrichment (gray trapezoids), the histone mark bound by HP1 via its chromodomain. Following DSBs, HP1 is evicted, allowing for Tip60 to interact with H3K9me3 through its own chromodomain. Tip60-dependant acetylation will favor chromatin remodeling and nucleosome removal, and probably resection, required for HR repair of DSB occurring in heterochromatin. (**C)** H4K20 mono and dimethylation (blue circles) are abundant modifications in mammalian genomes. Upon DSB, H4K20me1/2 is unmasked, via L3MBTL1 eviction or KDM4A degradation, DSB induced structural changes or histone modifications such as H2AK15ub (purple squares). This favors 53BP1 recruitment near the break sites, inhibits resection, allowing DSB repair by NHEJ.

Supporting the “chromatin driven DSB repair choice” hypothesis, we found that active genes are able to recruit the HR machinery thanks to the transcription-elongation associated H3K36me3 histone mark. Indeed, we showed that depletion of the main H3K36me3 histone methyltransferase, SETD2,^18^ is necessary for HR repair of “HR-prone” DSBs.^22^ Concomitantly, 2 other groups reported the critical function of human SETD2 in homologous recombination^23,24^ using other experimental systems such as I-SceI- and radiation-induced DSBs. Furthermore, SETD2 depletion seemed to favor repair by MMEJ.^24^ Importantly, neither SETD2 recruitment nor increased H3K36me3 levels were found at DSBs,^22-25^ suggesting that pre-established H3K36me3 channels active genes to HR repair. Notably, we found that RAD51 recruitment at HR-prone DSBs also depends on the lens epithelium-derived growth factor (LEDGF)/p75, which possesses a PWWP domain for H3K36me3-recognition. Since (LEDGF)/p75 also interacts with the resection promoting factor CtIP,^26^ we proposed a model where HR would be targeted at specific genomic locations by (LEDGF)/p75–mediated recruitment of CtIP on H3K36me3 enriched loci, such as actively transcribed genes (**Fig. 1A**). These findings are in good agreement with data from other labs showing that even in G2, the vast majority (roughly 85%) of irradiation or drugs induced DSBs are repaired by NHEJ,^10,27^ since active genes and H3K36me3 enriched loci represent only a minor fraction (few percent) of the genome.^28^

Studies in yeast have confirmed that Set2, the SETD2 homolog, also plays a key role in DNA repair pathway choice but in this case, Set2 favored NHEJ over HR.^29,30^ Those apparent discrepancies, discussed in,^31^ might arise from the fact that (i) LEDGF is not conserved in yeast, and (ii) Set2 mediates all 3 methylation states for H3K36^32^ while mammalian SETD2 mainly functions to convert H3K36me2 to H3K36me3.^18^ Indeed, interestingly, in mammals it appears that, in contrast to H3K36me3, the dimethylated form of H3K36 (H3K36me2), which is present on approximately 40% of nucleosomes ^33,34^ rather promotes NHEJ.^25^

## The“DSB Repair Choice Histone Code”

The above data thus demonstrate that along the chromosomes, all genomic loci are not repaired by the same pathway thanks to a chromatin dependent signaling, part of which relies on the methylation state of H3K36. It is likely that this concept could be expanded to other histone modifications and repair pathways. Indeed, many recent studies are in support of a specific histone code that favors, if not decides, on the pathway used to repair each specific genomic locus (**Fig. 1**).

First of all, DSBs that occur in heterochromatin are repaired by a specific HR pathway (dependent on ATM kinase, Artemis exonuclease, 53BP1, and the RNF168 and RNF8 ubiquitin ligases)^10,35,36^ The authors suggested that NHEJ might be inefficient to repair heterochromatic breaks due to the chromatin compaction observed in such regions. Consequent to NHEJ failure, heterochromatin decondensation occurs leading to recruitment of the HR machinery.^37^ Interestingly, these specific heterochromatic events could rely on a distinctive chromatin pathway involving HP1 (Heterochromatin Protein 1) and H3K9me3. Indeed, studies from B. Price's lab demonstrated that upon DSB induction, HP1 is evicted from chromatin, unmasking H3K9me3, the histone modification that HP1 usually binds. This provides a signal for the loading of the histone acetyl transferase (HAT) Tip60 that can interact with H3K9me3 via its chromodomain.^38^ HP1 and H3K9me3 are mainly located in heterochromatin and HAT recruitment has been proposed to facilitate nucleosome removal and resection. This mechanism could therefore specifically target HR at heterochromatic breaks (**Fig. 1B**). In addition, in mouse cells, H3K36me3 has also been found to be enriched in heterochromatic, DAPI-dense, regions,^39^ suggesting that H3K36me3 could promote HR repair at heterochromatic DSBs, similar to HR channeling at active genes.

Secondly, a fair amount of data describes the function of chromatin in the regulation of the 53BP1/BRCA1 axis. 53BP1 counteracts BRCA1 dependent resection, thus favoring NHEJ (for review^13,40^). 53BP1 harbors a tandem Tudor domain, that specifically recognizes H4K20me2 and H4K20me1 (and possibly with a lower affinity, H3K79me2).^41-45^ H4K20me2 is present on the vast majority of nucleosomes in the cell^46^ while H4K20me1 is found on coding region of actively transcribed genes.^47^ It is unclear whether or not H4K20 mono and/or dimethylation are efficiently induced at the vicinity of DSBs to recruit 53BP1.^43,48-52^ Interestingly, it has been suggested that DSB-induced alterations in chromatin structure could allow the specific unmasking of H4K20me1/2 at the vicinity of the break.^41,45^ Similarly, DSB-induced eviction of H4K20me2 binding proteins, such as L3MBTL1 and JMJD2A/KDM4A, would also permit unmasking of the preexisting H4K20me1/2 marks, allowing for damage-induced 53BP1 recruitment.^53,54^ Alternatively, 53BP1 might be recruited to damage by a combination of preexisting and DSB-induced histone marks, as shown for its dual recognition of H4K20me2 and H2AK15ub.^55^

Thus, similarly as with the situation observed for H3K36me3 mediated recruitment of (LEDGF)p75/CtIP, pre-existing H4K20me1/2 could help stabilize 53BP1 at DSBs, thus counteracting resection and favoring End Joining at genomic loci enriched in H4K20me1/2. In addition, a recent study revealed a role for acetylated H4K16 (H4K16ac), one of the histone marks associated with transcriptional activity, in counteracting 53BP1 binding to H4K20me2.^56^ Accordingly, H4K16ac enriched loci such as transcriptionally active genes, may be refractory to 53BP1 binding, thus allowing resection and RAD51 loading (**Fig. 1C**). Since all H4K16ac, H4K20me1 and H3K36me3 preferentially localize on genes, it will be of major interest now to investigate the interplay between these modifications and how they cooperate to regulate the 53BP1/BRCA1 balance and resection.

Notably, genetic studies in yeast led to the identification of a large number of histone residues required for genome stability.^57^ Since a growing number of proteins that harbor one or several histone modification binding modules (PHD, Tudor, PWWP, BRCT, chromodomain, bromodomain…) are recognized to be involved in the DNA damage response, it is tempting to extrapolate the aforementioned mechanisms in repair choice to other chromatin landscape and repair machineries. In agreement with this, SETD2 has been recently involved in facilitating mismatch repair (MMR),^58^ confirming the versatility of chromatin signaling in genome stability.

**Figure 2.**
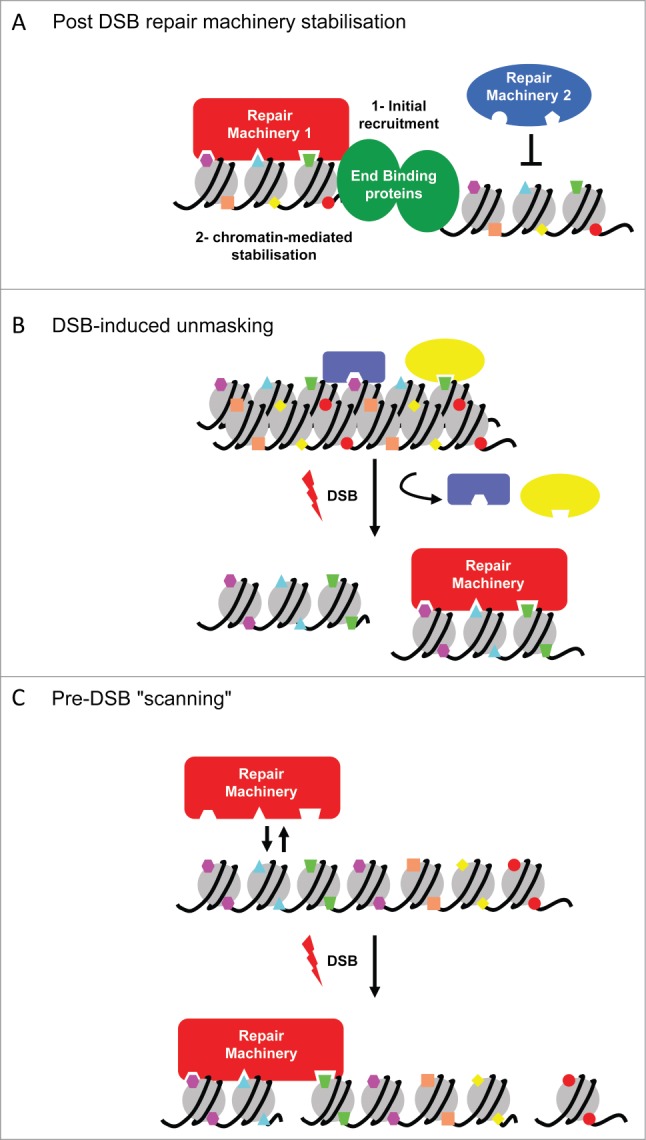
Potential mechanisms for preexistent chromatin structure in addressing repair pathways. (**A**) Following DSB and initial recognition by end binding proteins, such as Ku or the MRN complex, chromatin modifications already present at the break will stabilize component of a specific DNA repair pathway and/or inhibit association of component from another pathway. (**B**) DSB induces unmasking of existing histone marks by chromatin structure remodeling and/or chromatin reader eviction, making these modifications available for recognition by DNA repair factors. (**C**) In undamaged chromatin, repair proteins may preferentially interact with certain region of the genome based on their specific chromatin signature, facilitating their loading upon DSB induction.

An important issue resides in how can these histone modifications, already settled on chromatin before damage, recruit repair machineries only when a break occurs? First of all, initial recruitment of repair/signaling proteins could be mediated by end detection (by the Ku heterodimer, MRN complex and other DNA end binding proteins). Histone modifications, already available at the site of a break, would next stabilize or destabilize these machineries, helping the cell to fine tune the repair process at each DSB induced within the genome (**Fig. 2A**). Alternatively, as this was proposed for the recognition of H4K20me2 by 53BP1 and of H3K9me3 by TIP60 (see above), previously hidden histone modifications may be unmasked following DSBs (either by some topological changes induced by DSBs, or by protein removal) (**Fig. 2B**). Finally, in the absence of breaks, specific DNA repair proteins might also already be settled on chromatin or scanning certain region in the genome, as instructed by the appropriate chromatin signatures and be stabilized or activated upon the detection of a DSB (**Fig. 2C**).

## Concluding Remarks

The “chromatin-driven DSB repair pathway choice” could determine the repair accuracy and mutation rate taking place at each distinct locus in the genome. Genomes do not evolve homogenously. For example, while genes are highly conserved, some intergenic regions exhibit high mutation rates. These differences were generally attributed to 2 driving forces: a stronger selective pressure for coding (or regulatory) regions and an increased sensibility of certain loci to damaging agents or oncogenic stresses, defined as “fragile sites”. It is tempting to speculate that the inherent fidelity of the repair machinery toward each specific locus can also account for the variable mutation rates across the genome.

Even more importantly, chromatin can be directly modified in response to environmental inputs. This opens the possibility that external signals may fine tune repair accuracy at specific loci and therefore impact genome evolution and organism fitness. As a striking example, in yeast, nutrient availability regulates the Sir2 histone deacetylase, a key player in the regulation of rDNA copy number by regulating rDNA recombination (for review ^59^). That higher eukaryotic genome evolution might also be under the control of the environment, thanks to a fine tuning of DNA repair by a chromatin interface, represents an exciting area of future investigations.

## Disclosure of Potential Conflicts of Interest

No potential conflicts of interest were disclosed. 
